# Supplementary material for: Coupling of oceanic carbon and nitrogen facilitates spatially resolved quantitative reconstruction of nitrate inventories
Source: Nat Commun. 2018 Mar 23;9:1217. doi: 10.1038/s41467-018-03647-5 (PMC5865207; doi:10.1038/s41467-018-03647-5)
Supplement: Supplementary file 1 — Supplementary Information(PDF 2844 kb) [file 41467_2018_3647_MOESM1_ESM.pdf]

Supplementary Information for

"Coupling of oceanic carbon and nitrogen facilitates spatially resolved quantitative reconstruction of nitrate inventories"

by N. Glock *et al.*

Supplementary Note 1: The influence of microhabitat preferences on foraminiferal $\delta^{13}C$

Since *U. peregrina* lives shallow infaunal, an offset would be expected between $\delta^{13}C_{FORAM}$ in their tests and the $\delta^{13}C_{DIC}$ signature in the bottom water. This has been shown for *U. peregrina* specimens from the North Pacific and the Atlantic. The offset is strongly controlled by the gradient of $\delta^{13}C_{DIC}$ between bottom and pore waters[1]. The gradient of $\delta^{13}C_{DIC}$ between bottom and pore waters is controlled by bottom water oxygenation and diminishes with decreasing oxygen penetration depth into the sediments[2,3]. Since the Peruvian upwelling system is one of the most oxygen depleted regions in today´s ocean this gradient is supposed to be rather low at our sampling location. This might explain why there is no significant difference in the intercept between the downcore correlation of $[NO_3^-]_{BW}$ and $\delta^{13}C_{FORAM}$ and $[NO_3^-]$ and $\delta^{13}C_{DIC}$ in the recent water masses caused by vital effects. Nevertheless, *Virgulinella fragilis* from the anoxic Namibian diatomaceous mud belt show an extreme carbon fractionation of -12.5‰ probably derived from a mixture of carbon sources by anaerobic methane oxidation and enhanced incorporation of metabolic CO_2 derived from organic matter decomposition under oxygen depleted conditions[4]. For a comprehensive approach to reconstruct intermediate $[NO_3^-]$ using benthic $\delta^{13}C_{FORAM}$, it should be considered to focus on epifaunal species or to do a local species specific calibration to account for possible regional offsets between $\delta^{13}C_{FORAM}$ and bottom water $\delta^{13}C_{DIC}$ due to vital effects or changes within the microhabitat.

Supplementary Note 2: Local fluctuations of $[NO_3^-]_{BW}$

Local $[O_2]$ fluctuations are directly influencing NO_3^- loss processes at the Peruvian ODZ. As discussed in the main text, elevated $[O_2]$ during the LGM probably contributed to an increase in global $[NO_3^-]$ during the LGM. Since sediment core M77/2 52-2 is located in intermediate water depths well below the most oxygen depleted center of the ODZ a change in water column denitrification probably did not directly influence $[NO_3^-]_{BW}$ at this site. Benthic denitrification at depths below the Peruvian ODZ is negligible due to the lack of bacterial activity and low abundances of denitrifying foraminifera[5-7], probably related to the reduced C_{org} supply compared to the shelf sediments. The decrease in water column denitrification might locally lead to an increase in $[NO_3^-]$ but cannot explain a decrease in $\delta^{13}C_{DIC}$ which should be decoupled from denitrification. Although $[O_2]$ was probably increased during the LGM at the M77/2 52-2 site[8,9], a local decrease in denitrification cannot alone explain the tendencies observed in our sediment record, which follow to a major part the global changes in the oceanic $[NO_3^-]$ inventory.

Not all $[NO_3^-]_{BW}$ fluctuations in the record of core M77/2 52-2 can be explained by changes in the global reactive N reservoirs, though. The most well-marked offset to the global model predictions appears during H1, when $[NO_3^-]_{BW}$ depletes distinctively for ~4 kyrs (fig. 1B). The most important sources of nutrients in the Eastern Tropical Pacific are high latitude intermediate and deep waters. In the modern North Pacific deep waters are highly enriched in NO_3^- (see fig. 2A). During H1 a breakdown in stratification in the shallower North Pacific water masses has been observed[10]. This probably led to mixing of nutrient depleted $\delta^{13}C$ enriched surface water into intermediate and deep waters. Both, $[NO_3^-]_{BW}$ depletion and $\delta^{13}C$ enrichment, can be observed in the record of M77/2 52-2. On the contrary, there is evidence that Southern Ocean water masses were transported further northwards during this time interval than in the modern ocean, even towards the North Pacific[11]. Thus, it remains speculative if there was still a strong influence of North Pacific intermediate to deep water masses on the location of M77/2 52-2 in the Eastern Tropical South Pacific during H1. Nevertheless, the main nutrient supply

at this location remains the mixing between high latitude intermediate to deep waters. North Pacific water masses in the recent ocean show both higher $[NO_3^-]$ and lower $\delta^{13}C_{DIC}$ than the water masses in the Southern Ocean. Thus, both processes, a higher influence of Southern Ocean water masses and a depletion in nutrients in North Pacific intermediate waters by a breakdown in stratification, would lead to a depletion of nutrients in intermediate waters of the Eastern Tropical South Pacific.

Supplementary Note 3: The influence of anthropogenic CO_2 on $\delta^{13}C$

Penetration of anthropogenic CO_2 into the modern Pacific is already deeper than 2000 m[12,13]. The $\delta^{13}C$ values of anthropogenic CO_2 from the burning of fossil fuels, coal or natural gas are very low with a range from about -23.8‰ to -44.2‰[14]. At some stations in the modern Pacific the $\delta^{13}C_{DIC}$ signature is relatively low and shows a distinctive offset from the correlation between $\delta^{13}C$ and $[NO_3^-]$ which has been found in the modern Pacific as well as downcore over the last deglaciation. The deglacial correlation between $\delta^{13}C_{FORAM}$ and reconstructed $[NO_3^-]_{BW}$ is not influenced by anthropogenic CO_2. Since both linear regressions, for the modern Pacific and the downcore data (see Figure 2C and Supplementary Figure 4B), do not differ significantly from each other, the influence of anthropogenic CO_2 onto the correlation in eq.1 seems to be only of minor influence, yet. Offsets from the downcore correlation (Eq. S1), e.g. the distribution of stations with very low $\delta^{13}C_{DIC}$, might indeed be used to trace anthropogenic CO_2 in the modern Pacific.

Supplementary Eq. 1: $$\delta^{13}C = -0.115(\pm 0.015)\cdot[NO_3^-] + 4.520(\pm 0.634)$$

We calculated offsets from modern $\delta^{13}C_{DIC}$ and $\delta^{13}C_{DIC}$ predicted by the downcore correlation Supplementary Eq. 1 for the $[NO_3^-]$ measured at the same station. The distribution of these offsets ($\Delta\delta^{13}C$) at 700 m water depth of the modern Pacific is shown in Supplementary Figure 5. The distribution and quantity of negative $\Delta\delta^{13}C$ is directly reflection the distribution of anthropogenic CO_2 which has been found by previous modeling studies[12,13]. This indicates that Supplementary Eq. 1 might indeed be used to trace anthropogenic CO_2 and shows that a correlation which was even stable on glacial/interglacial timescales, although still consistent in most parts of the modern Pacific, already begins to break down at several locations which are strongly influenced by anthropogenic CO_2.

Supplementary Note 4: Offsets between measurements and modeling of the modern $\delta^{13}C_{DIC}$-$[NO_3^-]$-coupling

The model reproduces the modern observations in the intermediate Pacific Ocean reasonably well (R^2=0.638 , see Supplementary Figure 7). However, the model predicted $\delta^{13}C_{DIC}$-$[NO_3^-]$ slope (-0.066) is slightly lower than observed (-0.093) (Supplementary Figure 2). This underestimated slope is due to the too high $\delta^{13}C_{DIC}$ values in the deep North Pacific, the most notable bias in the modern model-data comparison (Supplementary Figure 7). This is caused by underestimated organic matter remineralization in the deep North Pacific that would further decrease $\delta^{13}C_{DIC}$ in the deep ocean. This slight systematic model bias likely applies to all time periods, which explains why the model predicts virtually no change to the $\delta^{13}C_{DIC}$-$[NO_3^-]$ slope between the modern and glacial ocean, particularly in the $[NO_3^-]$ range >40µM where our core location exists (Supplementary Figure 2b). This model result supports that the different environmental condition of the glacial ocean did not significantly change

the $\delta^{13}C_{DIC}$ -[NO_3^-] slope and that the observed modern ocean relationship is applicable in the glacial ocean.

Supplementary Table 3 shows the model NO_3^- sensitivity simulations when changing the amount of iron deposition to the Southern Ocean (LGM_lowSOFe, LGM_highSOFe). LGM_control simulates the Southern Ocean $\delta^{15}N_{sed.org}$ changes the best, suggesting it simulates the most realistic changes of enhanced remineralized [NO_3^-] in the deep ocean and reduced preformed [NO_3^-] in AAIW that affect our core location bottom water. The low and high Southern Ocean Fe simulation perform worse than the control simulation with respect to observations, but are within a reasonable uncertainty level considering the complexity of processes not directly tested here (see reference 15 for full discussion). These sensitivity simulations altered bottom water [NO_3^-] at our core location by an additional ±0.7 µM in addition to the change to global [NO_3^-], which can be considered as the uncertainty range of changes to our bottom water [NO_3^-] related to Southern Ocean iron fertilization that may not be reflecting global [NO_3^-] changes.

Supplementary Figures

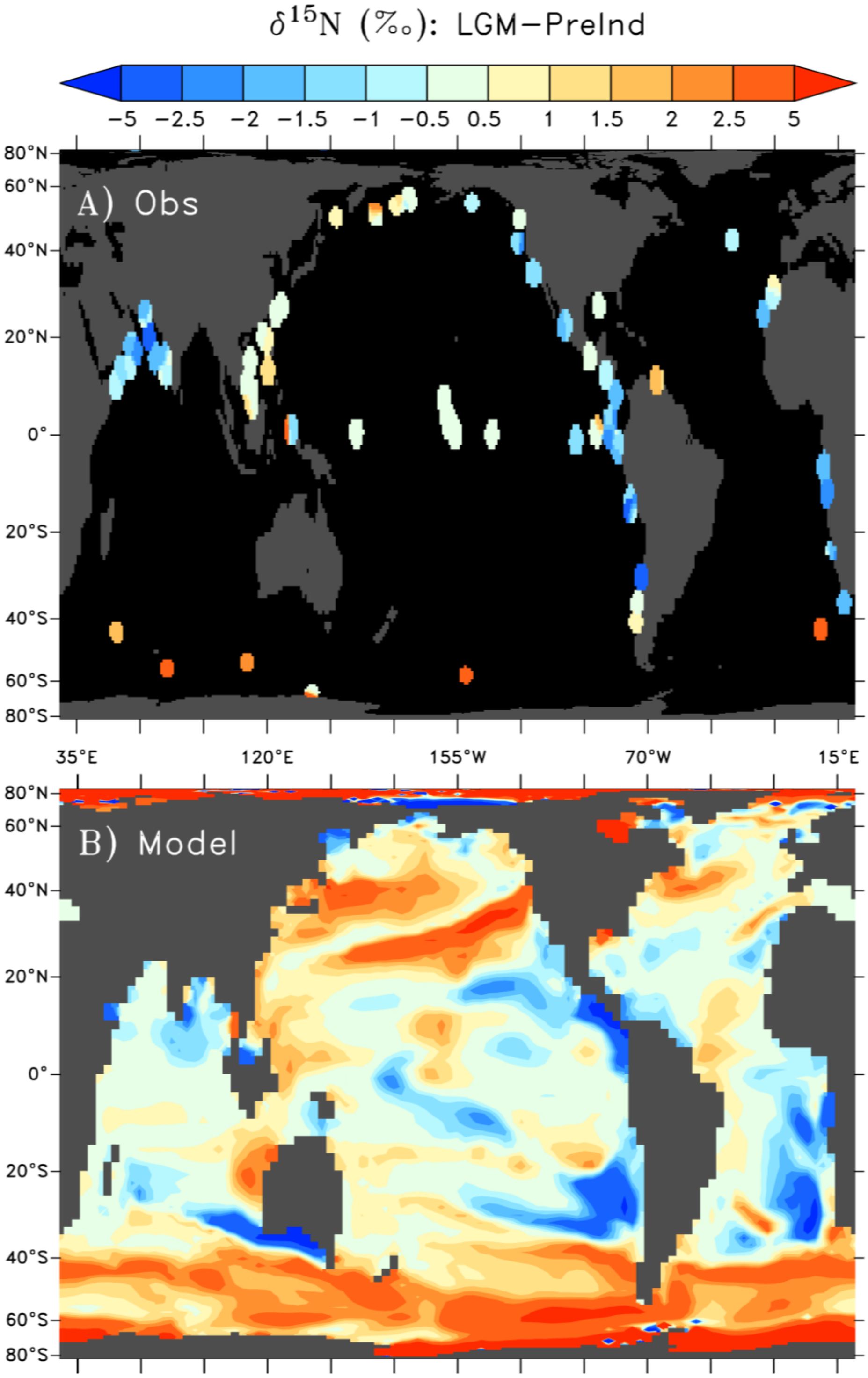

Supplementary Figure 1:
Model-data comparison of LGM minus pre-industrial global observations of $\delta^{15}N_{sed}$ with LGM_control and Pre-Industrial model simulations.

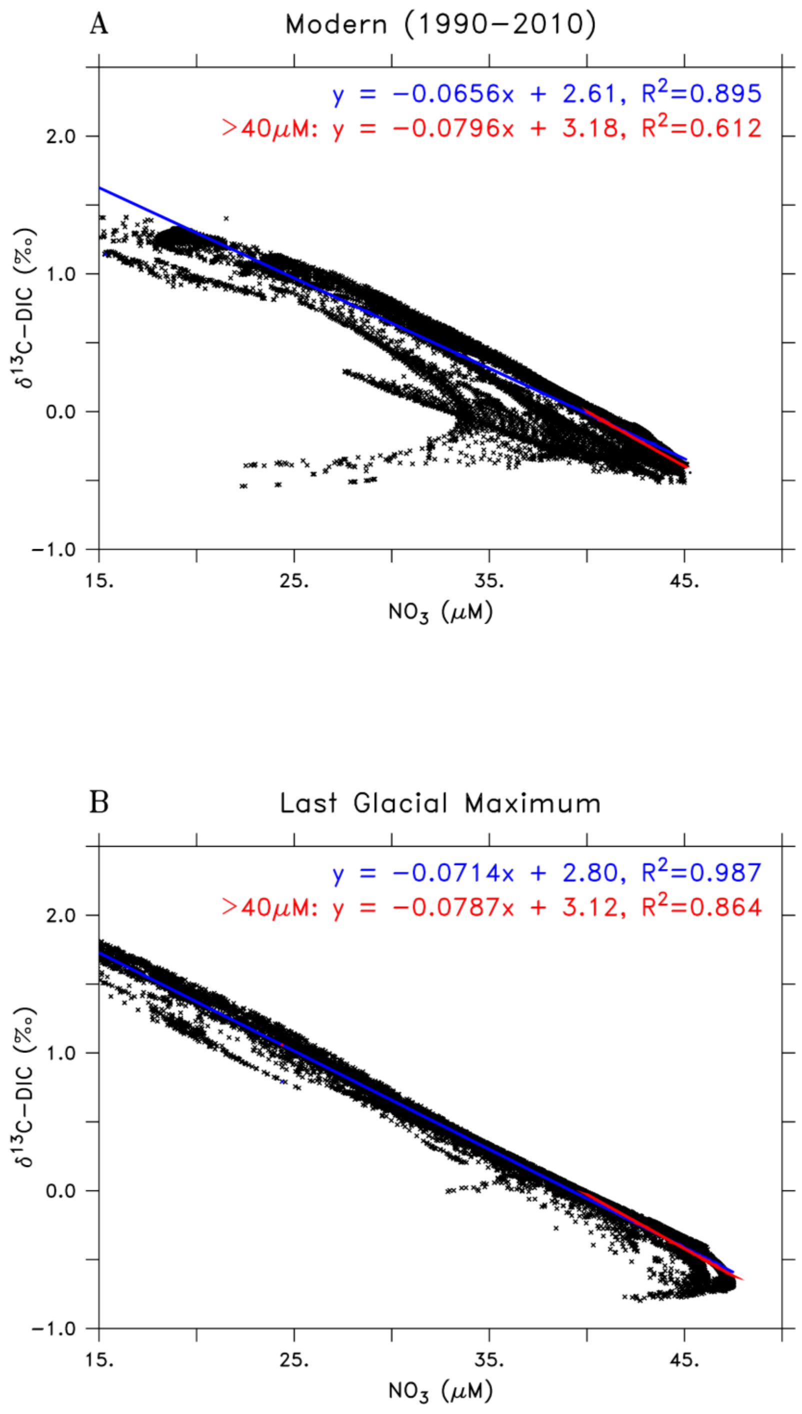

Supplementary Figure 2:
Model $\delta^{13}C_{DIC}$-[NO_3^-] relationship in the (A) modern ocean (1990-2010 average including anthropogenic effect of decreasing atmospheric $\delta^{13}CO_2$) and the (B) glacial ocean that applies model boundary conditions (i.e. greenhouse gases, insolation, ice sheets, iron deposition) from 21,000 years before present[15].

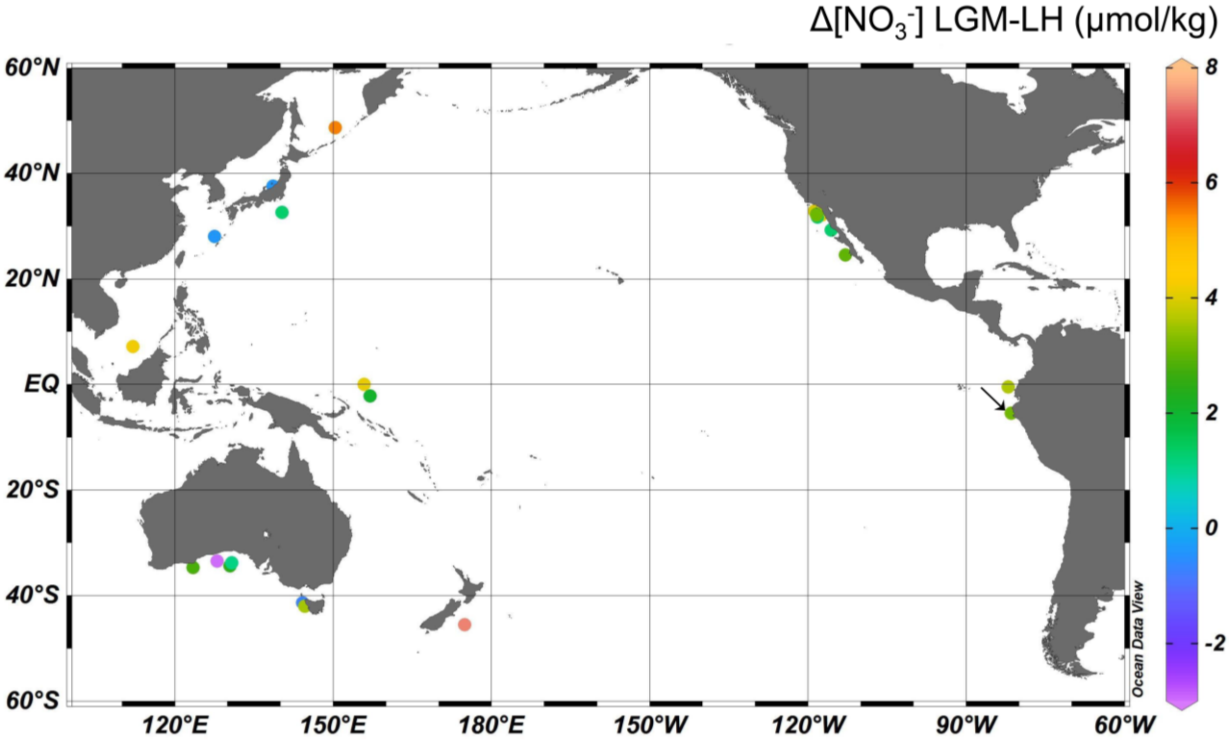

Supplementary Figure 3:
Regional reconstruction of the difference between the mean [NO_3^-] between the LGM (19 – 23 kyrs bp) and the Late Holocene (0-6 kyrs bp). Relative [NO_3^-] changes were calculated after equation 1 using the offset of mean $\delta^{13}C$ measured on *Cibicidoides spp.* between these two time intervals. Downcore $\delta^{13}C$ has been taken from supplementary references 16, 17 and 18. The station for which Δ[NO_3^-] has been reconstructed from the pore density of *Bolivina spissa* is marked with an arrow. The Ocean Data View software has been used to compile this plot[19].

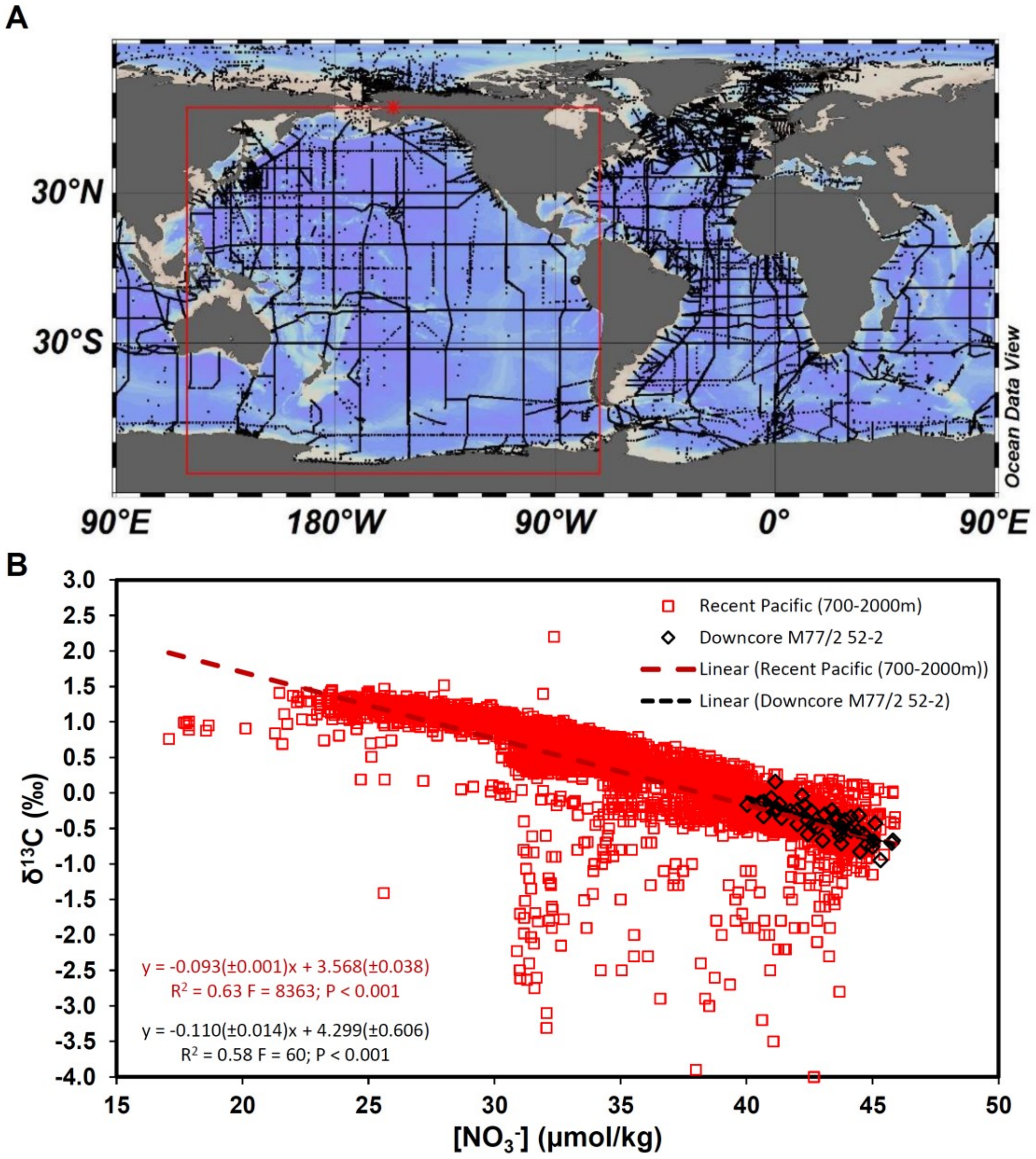

Supplementary Figure 4:

A: Overview map of all stations in the GLODAPv2 database[20]. The Ocean Data View software has been used to compile this plot[19].The red rectangular represents the boundaries of all stations included into the dataset which has been used for fig. 2C, fig. S2B and to calculate eq.1. **B:** Correlation between $[NO_3^-]$ and $\delta^{13}C$ in intermediate water depths (700-2000m) of the recent Pacific (red, N = 4779) and downcore of sediment core M77/2 52-2 (black, N = 44). Both linear regressions neither differ significantly in slope (P = 0.15) nor in intercept (P = 0.13). It is the same plot as shown in fig. 2C but not cut at a $\delta^{13}C$ of -1‰.

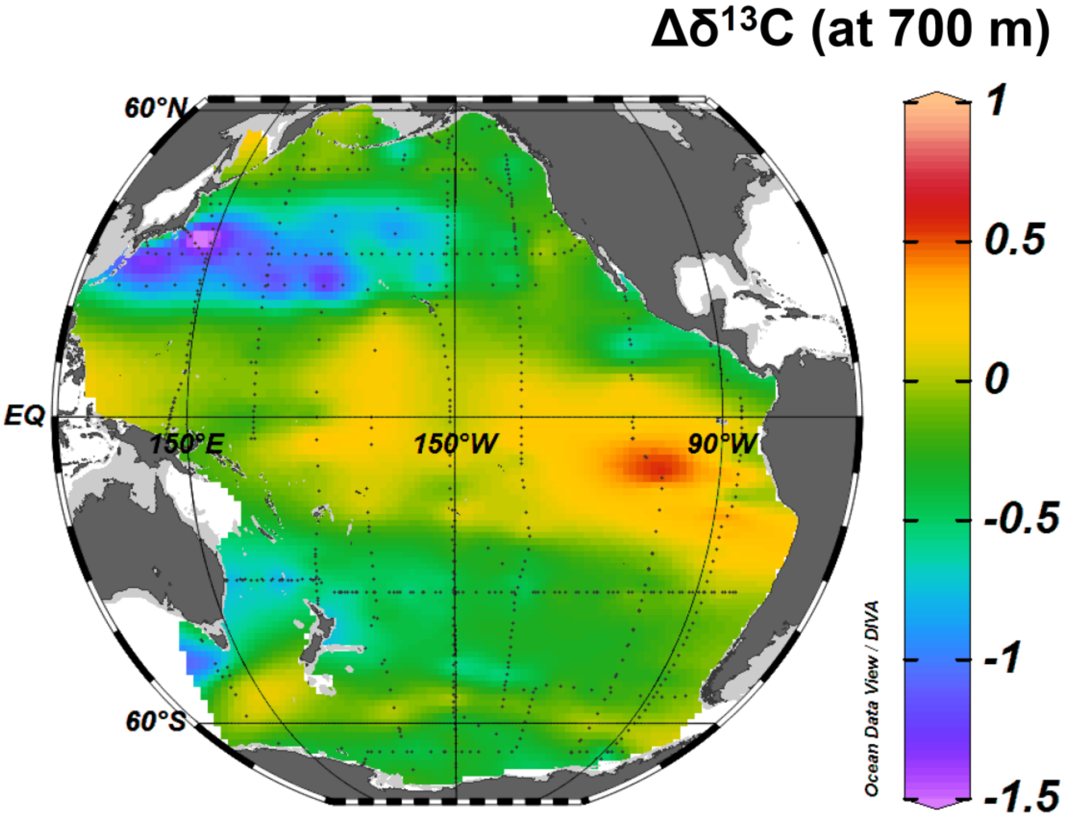

Supplementary Figure 5:
Offsets ($\Delta\delta^{13}C$) between $\delta^{13}C$ from DIC in the modern Pacific and $\delta^{13}C$ which would be predicted by Supplementary Eq. 1 for the measured [NO_3^-] at the same station. The distribution and quantity of negative $\Delta\delta^{13}C$ is directly reflecting the distribution of anthropogenic CO_2 in the modern Pacific which has been found in previous studies[12,13]. The Ocean Data View software has been used to compile this plot[19].

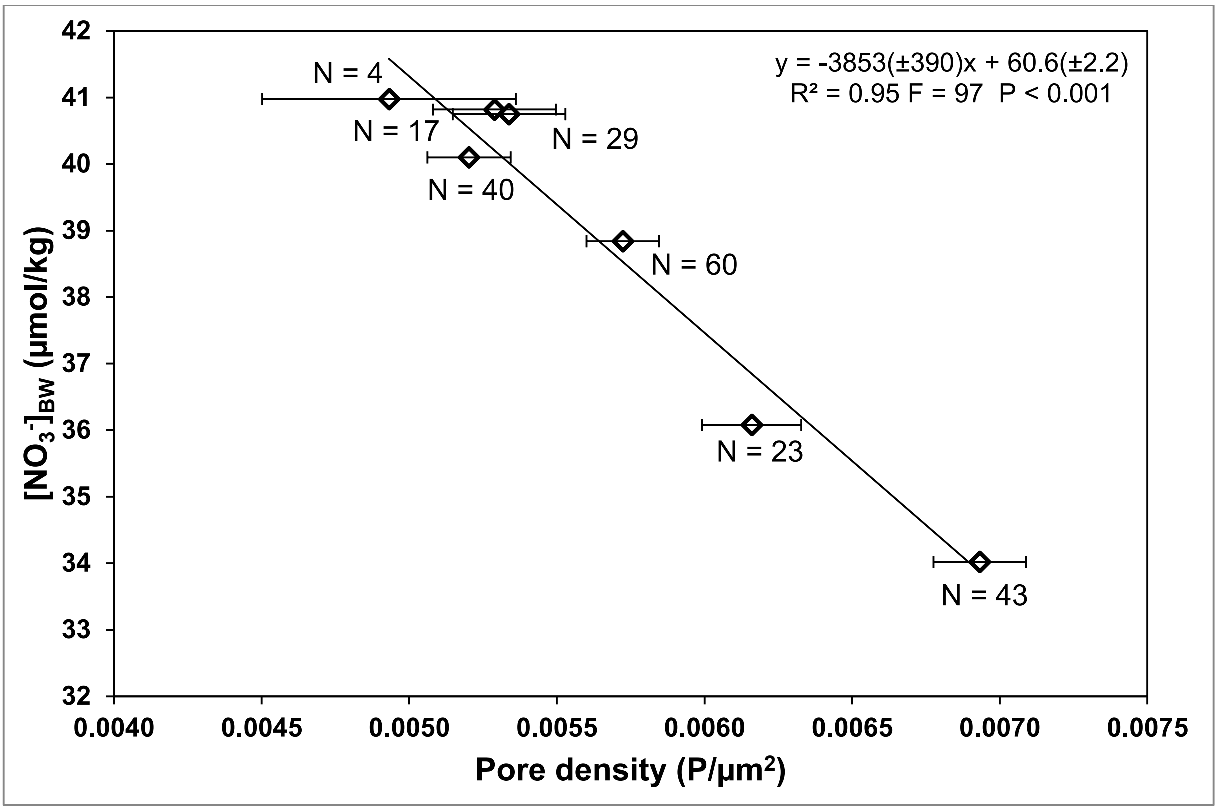

Supplementary Figure 6:
Correlations of *Bolivina spissa* pore density (PD) with $[NO_3^-]_{BW}$. PD of about the first 10 chambers (relates to an area of about 50,000–60,000 mm^2) is plotted. Error bars represent the standard error of the mean. The number of measurements for each location is indicated by N. Data has been taken from Supplementary Reference 21 figure 7D.

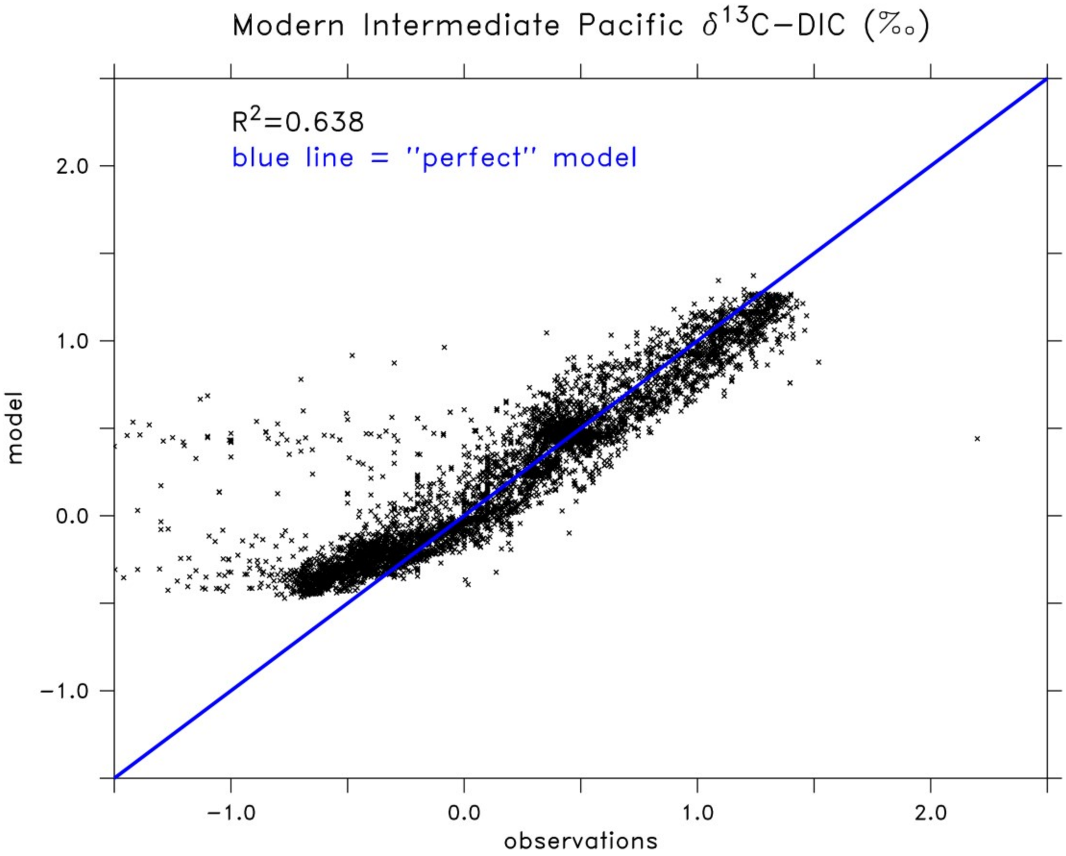

Supplementary Figure 7:
Model-data comparison of $\delta^{13}C_{DIC}$ in the intermediate Pacific (700-2000m).

Supplementary Tables

Supplementary Table 1: Downcore data for core M77/2 52-2. PD is the mean pore density of *Bolivina spissa* for this depth. N is the number of specimens used for the pore density analysis in this sample. $[NO_3^-]_{BW}$ was calculated from the pore density of *Bolivina spissa* using eq. 3. $\delta^{18}O$ and $\delta^{13}C$ were measured on *Uvigerina peregrina*. $\delta^{15}N$ was measured on bulk sediment.

Depth (cm)	Cal. Age (kyrs/BP)	PD (P/µm²)	N (#)	SEM_{PD} (P/µm²)	$[NO_3^-]_{BW}$ (µmol/kg)	SEM $[NO_3^-]_{BW}$ (µmol/kg)	$\delta^{18}O$ (‰)	$\delta^{13}C$ (‰)	$\delta^{15}N$ (‰)
10	0.3						3.27	-0.07	4.94
20	0.6	0.0048	20	0.0002	42.0	0.9	3.31	-0.24	
30	1.0						3.18	-0.26	4.79
40	1.3	0.0050	20	0.0002	41.2	1.0	3.15	-0.23	
50	1.7						3.08	-0.25	4.57
60	2.1	0.0047	20	0.0002	42.2	1.0	3.41	-0.04	
70	2.4	0.0051	20	0.0002	40.7	1.0	3.36	-0.33	4.55
80	2.8	0.0051	20	0.0002	40.7	1.0	3.37	-0.12	
90	3.1	0.0051	20	0.0002	41.0	0.9	3.23	-0.07	4.56
110	3.5	0.0044	17	0.0002	43.4	1.0	3.21	-0.24	4.50
120	3.8	0.0052	20	0.0002	40.5	1.1	3.26	-0.14	
130	4.2	0.0051	20	0.0002	40.9	1.0	3.24	-0.19	4.31
140	4.6	0.0050	20	0.0002	41.1	1.0	3.31	0.16	
150	4.9	0.0053	20	0.0003	40.0	1.3	3.29	-0.17	4.42
160	5.3	0.0050	20	0.0002	41.4	0.9	3.21	-0.35	
170	5.6	0.0047	20	0.0002	42.3	0.9	3.27	-0.17	4.51
180	6.0	0.0047	20	0.0003	42.6	1.2	3.28	-0.25	
190	6.3						3.22	-0.28	4.56
200	6.7	0.0050	20	0.0002	41.1	0.9	3.26	-0.25	
210	7.1	0.0050	18	0.0001	41.3	0.9	3.34	-0.15	4.79
220	7.4	0.0049	20	0.0002	41.7	0.9	3.28	-0.25	
230	7.8	0.0046	17	0.0002	42.6	1.2	3.06	-0.45	4.75
240	8.1	0.0047	21	0.0001	42.4	0.8	3.36	-0.38	
250	8.5	0.0042	20	0.0001	44.1	0.8	3.37	-0.34	4.96
260	8.8	0.0042	20	0.0001	44.5	0.8	3.50	-0.31	
270	9.2	0.0043	18	0.0002	43.9	0.9	3.49	-0.38	5.13
280	9.5	0.0044	20	0.0001	43.6	0.8	3.50	-0.52	
290	9.9	0.0044	20	0.0002	43.7	0.9	3.66	-0.60	5.25
300	10.3						3.55	-0.43	
310	10.6	0.0041	20	0.0002	44.6	0.8	3.44	-0.57	5.54
320	11.0						3.60	-0.54	5.31
330	11.3	0.0040	20	0.0002	45.1	1.0	3.66	-0.43	
340	11.8						3.81	-0.82	
350	12.3	0.0043	21	0.0002	43.8	1.0	4.05	-0.49	5.56
360	12.7	0.0042	21	0.0002	44.4	0.8	3.95	-0.54	
370	13.2						4.05	-0.69	5.81

380	13.6	0.0045	17	0.0001	43.1	0.9	4.04	-0.30	
390	14.1	0.0044	19	0.0001	43.6	0.8	4.14	-0.33	5.62
400	14.6	0.0044	20	0.0002	43.5	0.8	4.00	-0.36	
410	15.1	0.0048	17	0.0002	42.0	1.0	4.04	-0.44	5.01
420	15.6	0.0047	20	0.0002	42.4	1.1	4.08	-0.59	
430	16.2	0.0046	20	0.0002	42.8	1.0	4.18	-0.48	5.10
440	16.7	0.0042	20	0.0002	44.1	0.9	4.25	-0.53	
450	17.2	0.0041	19	0.0002	44.8	0.9	4.28	-0.72	5.63
460	17.7	0.0038	19	0.0001	45.8	0.8	4.34	-0.67	
470	18.2	0.0038	20	0.0001	45.8	0.8	4.30	-0.70	4.97
480	18.7						4.45	-0.45	
490	19.2	0.0040	20	0.0002	45.0	1.0	4.61	-0.76	4.36
500	19.7	0.0043	7	0.0003	43.8	1.5	4.44	-0.71	
510	20.2	0.0040	18	0.0001	45.0	0.9	4.76	-0.68	4.17
520	20.7	0.0042	7	0.0001	44.5	1.1	4.53	-0.83	
530	21.2	0.0039	19	0.0001	45.3	0.8	4.49	-0.95	4.03
540	21.7	0.0045	7	0.0004	43.0	1.8	4.74	-0.67	
550	22.2						4.48	-1.03	3.94
570	23.3								3.72
590	24.7								3.90

Supplementary Table 2: Model NO_3^- Sensitivity Results

	Core location NO_3^- (μM)	Global NO_3^- (μM)	SO surface NO_3^- (μM)	SO $\delta^{15}N_{bulk}$* (‰)
Pre-Industrial	40.4	30.5	18.0	3.10
Modern	40.3	30.4	17.5	3.26
LGM_control	43.3	34.9	16.7	5.54
LGM_lowSOFe	44.8	35.7	18.5	4.68
LGM_highSOFe	41.5	33.9	14.9	6.41

*The observed LGM-PI Southern Ocean (SO) $\delta^{15}N$ difference is 2.27‰, which supports that LGM_control (2.44‰) simulates the most realistic iron fertilization effect there, which is the simulation our main analysis is based upon, with LGM_lowSOFe (1.58‰) and LGM_highSOFe (3.31‰) representing our low- and high-end uncertainty sensitivity simulations.

Supplementary Table 3: All available records of δ^{13}C measured on *Cibicidoides spp.* from reference 16 available from intermediate water depths (700-2000m) within the longitude/latitude window defined in supplementary fig. S2A. Relative [NO_3^-] changes were calculated after eq. 1 (main manuscript) using the offset of mean δ^{13}C measured on *Cibicidoides spp.* between LGM (19 – 23 kyrs bp) and Late Holocene (0-6 kyrs bp). Stations which are written in **bold** letters represent the stations located in the Pacific which were used to calculate the mean Pacific deglacial [NO_3^-]. [NO_3^-]$_{BW}$ marked with a " * " was calculated using the pore density of *Bolivina spissa*. δ^{13}C at the station in *italic* letters was measured on *Uvigerina peregrina*. Superscripts at station names indicate reference numbers from additional literature.

Station name	Lat. (°N)	Long. (°W)	Depth (m)	δ^{13}C Hol (‰)	δ^{13}C LGM (‰)	[NO_3^-]$_{BW}$ Hol (µmol/kg)	[NO_3^-]$_{BW}$ LGM (µmol/kg)	Δ[NO_3^-]$_{BW}$ LGM-Hol (µmol/kg)
GL78-16	-76.0	-163.0	1937	1.07	NAN	26.7	NAN	NAN
ODP-1019	41.7	-124.9	860	NAN	0.07	NAN	37.4	NAN
BC87-21GC	23.3	-111.5	750	-0.20	NaN	40.3	NAN	NAN
BAP96-7T	25.7	-113.3	850	NAN	-0.10	NAN	39.3	NAN
CA94-12K-4	29.3	-115.6	863	NaN	-0.12	NAN	39.5	NAN
EW9504-09	32.9	-120.0	1194	NaN	-0.40	NAN	42.4	NAN
**EW9504-03**	**32.1**	**-117.4**	**1299**	**-0.22**	**-0.61**	**40.6**	**44.7**	**4.1**
**V19-27**	**-0.5**	**-82.1**	**1373**	**0.32**	**-0.01**	**34.7**	**38.3**	**3.6**
**EW9504-08**	**32.8**	**-118.8**	**1442**	**-0.09**	**-0.45**	**39.1**	**43.0**	**3.9**
**EW9504-04**	**32.3**	**-118.4**	**1759**	**-0.31**	**-0.38**	**41.5**	**42.3**	**0.7**
**ODP1012**	**32.3**	**-118.4**	**1783**	**0.08**	**-0.20**	**37.3**	**40.3**	**3.0**
**AHF16832**	**31.7**	**-118.2**	**1915**	**-0.31**	**-0.43**	**41.5**	**42.8**	**1.3**
V35-05	7.2	112.1	1953	0.01	-0.38	38.1	42.3	4.2
BO94-20 PN3PC	28.1	127.6	1058	0.07	0.11	37.4	37.0	-0.4
RS102-GC09	-33.5	128.0	769	1.45	1.73	22.7	19.7	-3.0
KH82-4-14	31.4	129.0	740	NAN	0.38	NAN	34.1	NAN
RS102-GC14	-34.4	130.4	1502	0.66	0.43	31.1	33.6	2.5
RS102-GC13	-33.8	130.8	1008	1.11	1.01	26.3	27.4	1.1
RS67-GC3	37.6	138.6	1476	0.45	0.49	33.4	32.9	-0.4
RS67-GC16	38.3	140.2	1650	NAN	0.21	NAN	35.9	NAN
V18-222	38.5	140.2	1904	NAN	-0.04	NAN	38.6	NAN
**V20-133**	**32.6**	**140.3**	**1503**	**-0.07**	**-0.19**	**38.9**	**40.2**	**1.3**
RS67-GC52	41.2	144.0	1145	NAN	-0.03	NAN	38.5	NAN
RS78-GC18	-41.4	144.2	814	1.13	1.20	26.1	25.3	-0.7
**Nes-BC-32**	53.6	144.6	1000	-0.16	NAN	39.9	NAN	NAN
SO36-SL17	-42.1	144.6	1042	1.03	0.70	27.2	30.7	3.5
SO36-SL7	-42.3	144.7	1085	NAN	0.66	NAN	31.1	NAN
V32-161	48.3	149.1	1600	NAN	-0.36	NAN	42.0	NAN
Nes-GGC-27	49.6	150.2	995	NAN	0.36	NAN	34.3	NAN
B34-19	49.1	150.3	1227	NAN	0.11	NAN	37.0	NAN
Nes-GGC-20	48.9	150.4	1510	NAN	-0.11	NAN	39.4	NAN

Nes-GGC-18	48.8	150.4	1700	NAN	-0.14	NAN	39.7	NAN
Nes-GGC-15	48.6	150.4	1980	0.10	-0.41	37.1	42.6	5.5
**ERDC88bx**	**0.0**	**155.9**	**1924**	**0.42**	**0.04**	**33.7**	**37.8**	**4.1**
**ERDC92bx**	**-2.2**	**157.0**	**1598**	**0.41**	**0.22**	**33.8**	**35.8**	**2.0**
**MD97-2120**	**-45.5**	**174.9**	**1210**	**0.98**	**0.29**	**27.7**	**35.1**	**7.4**
M77/2 52-2	*-5.5*	*-81.5*	*1250*	*-0.17*	*-0.76*	*41.0**	*44.1**	*3.1**
**CA94-12K-4**[17]	**29.3**	**-115.6**	**860**	**0.00**	**-0.12**	**38.2**	**39.5**	**1.3**
**ET97-4T**[18]	**24.5**	**-112.9**	**1450**	**0.10**	**-0.18**	**37.1**	**40.1**	**3.0**
**EW95-04**[18]	**32.1**	**-118.4**	**1765**	**0.09**	**-0.2**	**37.2**	**40.3**	**3.1**
**MD03-2614G**[18]	**-34.7**	**123.4**	**1070**	**1.00**	**0.74**	**27.5**	**30.3**	**2.8**

Supplementary References:

1. McCorkle, D. C., Corliss, B. H., & Farnham, C. A., Vertical distributions and stable isotopic compositions of live (stained) benthic foraminifera from the North Carolina and California continental margins, *Deep Sea Res. I* **44**, 983, 1997.

2. McCorkle, D. C., Emerson, S. R. and Quay, P. D., Stable carbon isotopes in marine porewater, *Earth Plan. Sci. Let.* **74**, 13-26, 1985.

3. Hoogakker, B. A. A. *et al.*, Glacial–interglacial changes in bottom-water oxygen content on the Portuguese margin, *Nat. Geosci*. **8**, 40-43, 2015.

4. Altenbach, A.V. *et al.* Carbon and Nitrogen isotopic fractionation in foraminifera: Possible signatures from anoxia, in ANOXIA: Evidence for eukaryote survival and paleontological strategies, Cellular Origin, Life in Extreme Habitats and Astrobiology 21, edited by: Altenbach, A. V., Bernhard, J. M., and Seckbach, J., Springer Science+Business Media, 2011, 519–535, 28, 2012.

5. Glock, N., *et al.*, The role of benthic foraminifera in the benthic nitrogen cycle of the Peruvian oxygen minimum zone, *Biogeosciences* **10**, 4767–4783, 2013.

6. Dale, A. W., *et al.*, Biological nitrate transport in sediments on the Peruvian margin mitigates benthic sulfide emissions and drives pelagic N loss during stagnation events, *Deep Sea Res. I* **112**, 123-136, 2016.

7. Sommer, S., *et al.*, Depletion of oxygen, nitrate and nitrite in the Peruvian oxygen minimum zone cause an imbalance of benthic nitrogen fluxes, *Deep Sea Res. I* **112**, 113-122, 2016.

8. Moffitt , S.E., *et al.*, Paleoceanographic insights on recent oxygen minimum zone expansion: lessons for modern oceanography, *PLoS One* **10**, 2015.

9. Salvatteci, R., *et al.,* Centennial to millennial-scale changes in oxygenation and productivity in the Eastern Tropical South Pacific during the last 25,000 years, *Quat. Sci. Rev.* **131**, 102-117, 2016.

10. Rae, J. W. B., *et al.* Deep water formation in the North Pacific and deglacial CO_2 rise, *Paleoceanography* **29**, 645–667, 2014.

11. Basak, C., *et al.* Southern Ocean source of ^{14}C-depleted carbon in the North Pacific Ocean during the last deglaciation, *Nat. Geosci.* **3**, 770-773, 2010.

12. Chen, A., Anthropogenic CO_2 distribution in the North Pacific Ocean, *J. Oceanogr.* **49**, 257-270, 1993.

13. Sabine, C. *et al.* Distribution of anthropogenic CO_2 in the Pacific Ocean, *Global Biogeochem. Cycles.* **16**, 1083, 2002.

14. Pugliese, S. *et al.* Characterization of the $\delta^{13}C$ signatures of anthropogenic CO_2 emissions in the Greater Toronto Area, Canada, *Ap. Geochem.* Available online 9 November 2016.

15. Somes, C. *et al.* A Three-Dimensional Model of the Marine Nitrogen Cycle during the Last Glacial Maximum Constrained by Sedimentary Isotopes. *Front. Mar. Sci.* **4***,* 108*,* 2017.

16. Petersen, C. D., Lisiecki, L. E. and Stern, J. V., Deglacial whole-ocean δ13C change estimated from 480 benthic foraminiferal records, *Paleoceanography* **29**, 549-563, 2014.

17. van der Kaars, S. *et al.*, Humans rather than climate the primary cause of Pleistocene megafaunal extinction in Australia, *Nat. Comm.*, 8:14142, 2017.

18. Herguera, J., Intermediate and deep water mass distribution in the Pacific during the Last Glacial Maximum inferred from oxygen and carbon stable isotopes, *Quat. Sci. Rev.*, **29**, 1228-1245, 2010.

19. Schlitzer, R., Ocean Data View, http://odv.awi.de, 2015.

20. Olsen, A. *et al.* The Global Ocean Data Analysis Project version 2 (GLODAPv2) - an internally consistent data product for the world ocean, *Earth System Science Data*, **8**, 297-323, 2016.

21. Glock, N. *et al.* Environmental influences on the pore-density in tests of *Bolivina spissa*. *J. Foramin. Res.* **41**, 22–32, 2011.
